# Do Depressive Symptoms Shape Blacks’ Perceptions of Stress Over Time?

**DOI:** 10.1093/geroni/igaa057.1901

**Published:** 2020-12-16

**Authors:** DeAnnah Byrd, Roland Thorpe, Keith Whitfield

**Affiliations:** 1 Wayne State University, Detroit, Michigan, United States; 2 Johns Hopkins Bloomberg School of Public Health, Baltimore, Maryland, United States

## Abstract

The established association between stress and depression is typically examined only in one direction and cross-sectionally. Data from the Baltimore Study of Black Aging-Patterns of Cognitive Aging was used to longitudinally examine the bi-directional relationships between (1) stress-depression and (2) depression-stress, and age as a modifier. The sample consisted of 602 community-dwelling Blacks, aged 48-92 years at baseline and 450 at follow-up 33 months later. While the stress-depression relationship was non-significant; the depression-stress was (b= 0.236, p< 0.000) and this association varied by age with the impact of baseline depression on changes in stress greatest among Blacks in their 60’s versus those in their 50’s (b= 0.257, p= 0.002), controlling for model covariates. Findings highlight the importance of depression in shaping Blacks’ perception of stress over time. Future work should continue to identify stress and mental health risk factors that contribute to poor health and health disparities in older Blacks.

